# Modern Problems in Psychiatry

**Published:** 1914-06

**Authors:** 


**Modern Problems in Psychiatry.** By ERNESTO LUGARO. Translated by DAVID ORR, M.D., and R. G. ROWS, M.D. Pp. vii, 305. Manchester: At the University Press. 1913. Price, 7s. 6d. net.

We must at the outset commend the admirable translation by Drs. Orr and Rows. To them we owe an eminently clear and polished version of a most valuable work. Lugaro has in this second edition laid under review all reliable research work up to date. That he has been able to compress, without evidence of undue brevity so large a bulk of material within such reasonable limits, is owing to his powers of complete assimilation and comprehension, to the filtration to essentials accomplished by a mind clear, critical and unbiassed. His statements, indeed, may be absorbed by the reader with little hesitation: they have passed through an exceptionally keen scrutiny before presentment. After the perusal of this work one may feel that reproach can no longer be cast on psychiatry as a laggard branch of medicine. It is evident, although but the fringe of the fabric be shown, that considerable and reliable additions to the structure have been made of late years. To none more than the author does credit belong for such advances, and though

Lugaro is ruthless in exhibiting the shortcomings in our knowledge, his candour is the more justifiable by reason of the realisation of firmer ground and the obvious need for filling up the now recognisable voids. At the same time, he is full of hope and of suggestions of fruitful kind for the lines of future investigations. Any detailed notice of this book is rendered superfluous by the clear and appreciative summary contained in the Foreword by Sir Thomas Clouston; its main features, too, have received allusion in the notice of the first edition. It is to be recommended with confidence to the general physician and neurologist as well as to the alienist. To the latter it is indispensable should he desire to keep abreast of recent research. It is rare that such a summary of the world's work can be presented in so assimilable and impeccable a form, and with such a blending of original investigation and valuable comment.

